# Exosomes combined with biomaterials in the treatment of spinal cord injury

**DOI:** 10.3389/fbioe.2023.1077825

**Published:** 2023-03-13

**Authors:** Xuanxuan Zhang, Wenwei Jiang, Yan Lu, Tiantian Mao, Yu Gu, Dingyue Ju, Chuanming Dong

**Affiliations:** Department of Anatomy, Medical College of Nantong University, Nantong, China

**Keywords:** spinal cord injury, exosomes, biomaterial scaffold, stem cells, axonal regeneration

## Abstract

Spinal cord injury (SCI) is a serious and disabling disease with a high mortality rate. It often leads to complete or partial sensory and motor dysfunction and is accompanied by a series of secondary outcomes, such as pressure sores, pulmonary infections, deep vein thrombosis in the lower extremities, urinary tract infections, and autonomic dysfunction. Currently, the main treatments for SCI include surgical decompression, drug therapy, and postoperative rehabilitation. Studies have shown that cell therapy plays a beneficial role in the treatment of SCI. Nonetheless, there is controversy regarding the therapeutic effect of cell transplantation in SCI models. Meanwhile exosomes, as a new therapeutic medium for regenerative medicine, possess the advantages of small size, low immunogenicity, and the ability to cross the blood-spinal cord barrier. Certain studies have shown that stem cell-derived exosomes have anti-inflammatory effects and can play an irreplaceable role in the treatment of SCI. In this case, it is difficult for a single treatment method to play an effective role in the repair of neural tissue after SCI. The combination of biomaterial scaffolds and exosomes can better transfer and fix exosomes to the injury site and improve their survival rate. This paper first reviews the current research status of stem cell-derived exosomes and biomaterial scaffolds in the treatment of SCI respectively, and then describes the application of exosomes combined with biomaterial scaffolds in the treatment of SCI, as well as the challenges and prospects.

## 1 Introduction

Spinal cord injury (SCI) is a severely devastating neurological injury that can result in complete or incomplete loss of voluntary motor function and sensory dysfunction, and have a high risk of complications. SCI is followed by a series of secondary pathophysiological changes that induce an inflammatory response and neuronal apoptosis at the site of the injury, followed by cavity formation and astrocytic scars, leading to the inhibition of axonal regeneration (Wertheim et al., 2022). According to the National Center for Spinal Cord Injury Statistics Facts and Figures 2020, there are approximately 294,000 new cases of SCI per year worldwide (Shen et al., 2021). Primarily, treatment of SCI includes spine immobilization, surgical decompression, and postoperative rehabilitation (Shen et al., 2021), however, these approaches have shown limited effectiveness in clinical practice.

**FIGURE 1:**
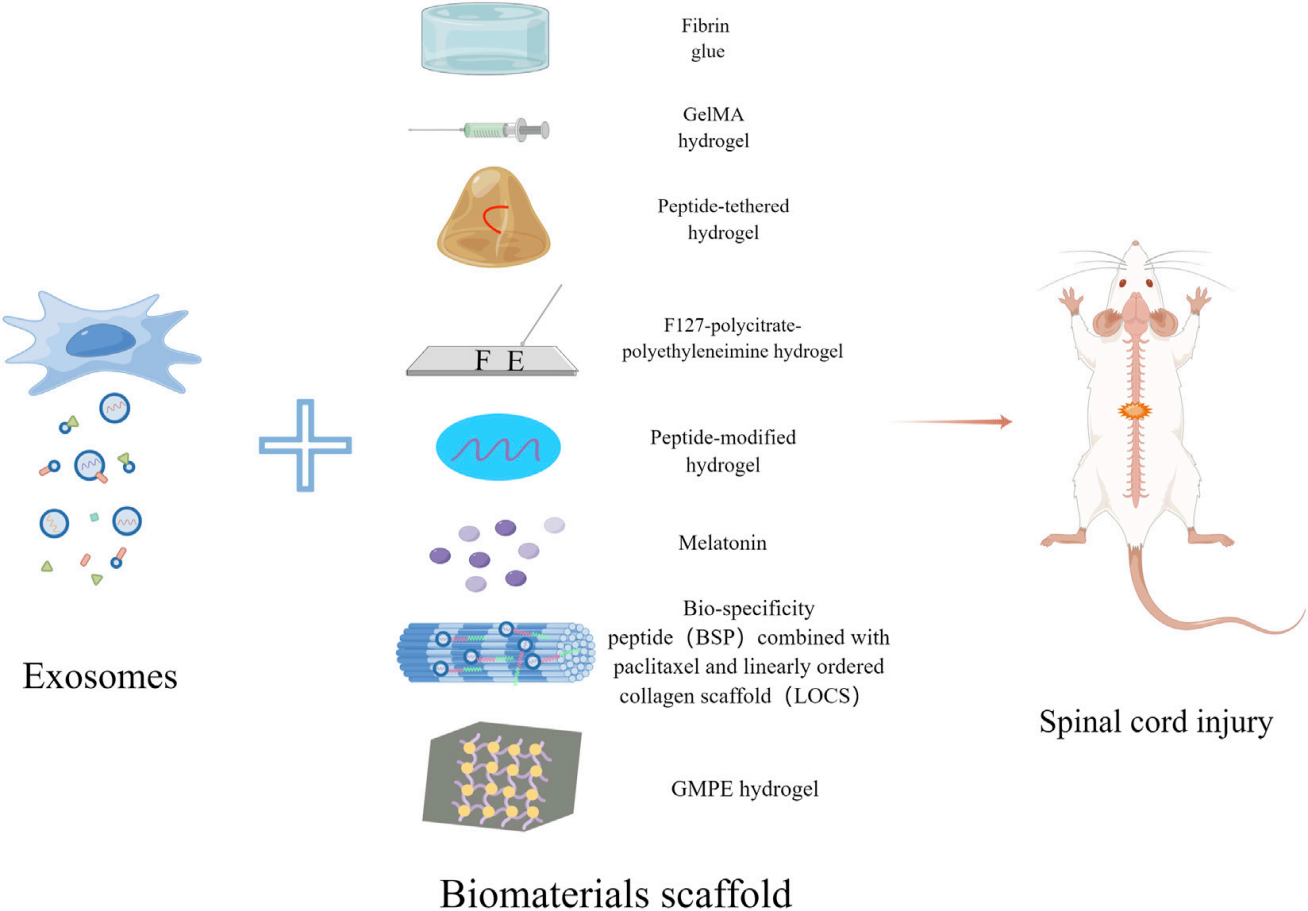
Schematic diagram of exosomes combined with biomaterials for the treatment of SCI. Exosomes derived from stem cells can be combined with Fibrin glue, GelMA hydrogel, Peptide-tethered hydrogel, F127-polycitrate-polyethyleneimine hydrogel, Peptide-modified hydrogel, Melatonin, LOCS-BSP and GMPE hydrogel respectively to treat spinal cord injury.

Cell transplantation offers a new treatment option for SCI, and numerous studies have proved their effectiveness. So far, a large number of cells have been used in SCI models for treatment, such as mesenchymal stem cells (MSCs), neural stem cells (NSCs), embryonic stem cells, olfactory ensheathing cells, and Schwann cells (SCs). However, compared to cells, exosomes secreted by cells have advantages such as neuroprotection, blocks apoptosis, low immunogenicity, easy storage and transport, axonal regeneration, small size that is not captured by lung and liver tissues, and the ability to cross the blood-spinal cord barrier (Khalatbary, 2021). The function of exosomes also depends on the type of cells from which they originate, their status and their environment. The development of tissue engineering techniques has provided new ideas for the treatment of SCI by inoculating exosomes onto biomaterial scaffolds, which provide the physical structure for exosome growth and differentiation and promote the regeneration of residual neuronal axons. At the same time, the scaffold needs to meet certain requirements: firstly, it should have the appropriate softness and certain water retention; secondly, it should have good biodegradability and non-toxicity; in addition, the biomaterial should have certain porosity and mechanical strength. Currently, there are three main forms of biomaterial scaffolds for SCI: hydrogels, 3D printing and nanomaterials (Liu et al., 2019). Biomaterials can be used to load exosomes and provide appropriate nutritional factors to the SCI site (illustrated in Figure 1).

In this paper, we review the different sources of exosomes and various biomaterials currently used for spinal cord injury, and we describe the progress of the combination of exosomes and biomaterials for the treatment of SCI.

## 2 Pathophysiology of SCI

SCI is mainly caused by a primary insult followed by secondary injuries. Primary injuries mostly originate from car accidents, falling, and sports injury, among others. These injuries are irreversible and can cause a series of neurological symptoms such as sensorimotor dysfunction, autonomic dysfunction, urinary and genital infections, and pressure sores depending on the site and severity of the injury (Katoh et al., 2019). Secondary injury is a pathological process caused after the primary injury that includes continuous hemorrhage, neuroinflammation, oxidative stress, and edema leading to massive necrosis, which in turn trigger free radical formation, glutamate-mediated excitotoxicity, and neurotoxicity (Anjum et al., 2020). Further, reactive astrocytes and oligodendrocyte precursor cells were activated to secrete chondroitin sulfate proteoglycans (CSPGs), causing glial scar formation, which formed a physical barrier to inhibit axonal growth (Yang et al., 2020). Therefore, the treatment for SCI should focus on how to promote axonal regeneration, reduce inflammation, and remodel functional neural circuit.

## 3 Cell-derived exosomes

Due to the lack of human pharmacological treatment options, rat and mouse models of SCI are commonly used for innovative research. Methylprednisolone, melatonin and riluzole are currently being tested in humans and animals, but their effectiveness is still controversial. For instance, adverse effects have been shown in several studies, including pancreatitis, lung disease, and neutropenia (Srinivas et al., 2019). In this context, many new treatments for SCI are being developed (Yi and Wang, 2021). Currently, there are many different types of cell transplantation strategies used in therapeutic studies of SCI, including NSCs, MSCs, olfactory ensheathing cells, and SCs (Feng et al., 2021). However, cell therapy for spinal cord injury may have the following disadvantages: most of the cells transplanted intravenously pooled at the lung and liver and could not fully exploit their effects at the site of SCI (Lu et al., 2019). The SCI pathological microenvironment inhibits the survival of transplanted cells (Liu W. Z. et al., 2021). Stem cells may have potential tumorigenic risk and ethical concerns (Sun et al., 2018).

There are numerous studies that suggest that the mechanism of stem cell therapy for SCI is closely related to their paracrine effects (Zhang et al., 2022). In recent years, exosomes have been recognized as paracrine factors released by almost all types of cells (Romanelli et al., 2019). Exosomes are vesicles that are continuously released by cells to the extracellular environment, with a diameter of about 40–100 nm, and contain a variety of biomolecules such as lipids, proteins and nucleic acids (He et al., 2018). Extracellular vesicles can be divided into exosomes, apoptotic body, and vesicles based on the release mechanism and size, with exosomes having the smallest diameter (Zhang et al., 2022). Exosomes can be detected in all body fluids, including urine, blood and cerebrospinal fluid (Dutta et al., 2021). Exosomes are neuroprotective, angiogenic, and immunomodulatory, and easily cross the blood-spinal cord barrier and therefore can promote axonal regeneration (Yang et al., 2020). In addition, they have been found to inhibit the activation of M1 microglia and type A1 astrocytes and reduce neuroinflammation (Jiang et al., 2020; Khalatbary, 2021). At the same time, exosome treatment does not carry the same tumorigenic risks associated with cell transplantation and as they are not easily captured by lung and liver tissue due to their small size.

One of the challenges hindering the clinical translation of exosomes is the lack of standard methods for isolation and purification. Currently, ultracentrifugation is the most commonly used isolation method; however, as exosomes are precipitates obtained by multiple centrifugations and filtrations, isolation of non-vesicular entities from exosomes has not been fully achieved, and there is no method that can achieve both high recovery and specificity of the isolated exosomes (Guo et al., 2021).

### 3.1 MSC-derived exosomes (MSCs-Exos)

MSCs are multipotent mesenchymal stromal cells with self-renewal capacity and neuroprotective capability that promote tissue repair. They can be obtained from tissues such as bone marrow, umbilical cord, dental pulp, menstrual blood, and fat, and can be isolated from autologous or allogeneic sources (Lopez-Verrilli et al., 2016; Sykova et al., 2021). MSCs possess a variety of growth factors and cytokines, and have anti-inflammatory effects, which are valuable in the treatment of SCI, but there are still some limitations when directly transplanting MSCs into target tissues (Kim et al., 2021; Liu W. Z. et al., 2021). Due to the presence of the blood-spinal cord barrier, transplanted MSCs rarely reach the site of injury after intravenous injection. Recent studies suggest that the role of MSCs in the treatment of SCI is attributed to their paracrine mechanism and that exosomes may play an important role in this process (Lee et al., 2020; Liu et al., 2020). Angiogenesis is crucial in the repair after SCI, and MSCs-Exos promotes the repair of injured spinal cord tissue by inducing angiogenesis, modulating immune and inflammatory responses, inhibiting apoptosis (Zhao et al., 2019), reducing CSPGs, and maintaining the integrity of the blood-spinal cord barrier (Ren et al., 2020). After treatment with MSCs-Exos, the expression levels of pro-apoptotic proteins (Bax) and pro-inflammatory cytokines (TNF-α and IL-β) were significantly reduced, while the expression levels of anti-apoptotic (Bcl2) and anti-inflammatory (IL-10) proteins were increased (Huang et al., 2017). Finally, the immune system does not perceive exosomes as foreign bodies, making them more suitable for clinical application than stem cells (Ghafouri-Fard et al., 2021).

#### 3.1.1 Bone marrow mesenchymal stem cell-derived exosomes (BMSCs-Exos)

Studies have shown that human BMSCs-Exos are commonly available and can play a good neuroprotective role in the treatment of SCI (Chang et al., 2021). The blood-spinal cord barrier has an important role in functional recovery after SCI, helping to regulate the entry of external substances into the central nervous system and maintaining the homeostasis of the microenvironment. Disruption of the blood-spinal cord barrier leads to neurological deficits by infiltration of inflammatory substances, production of neurotoxic substances, and disruption of neuronal and synaptic function (Xin et al., 2021). Pericytes are a central component of the neurovascular unit, separated from endothelial cells, and are important for regulating blood-spinal cord barrier permeability, capillary blood flow, and immune cell entry into the central nervous system (CNS). BMSC-Exos reverse blood-spinal cord barrier leakage and reduce edema, which leads to improved pericyte coverage and better functional recovery after injury (Lu et al., 2019; Nakazaki et al., 2021; Zhou et al., 2022). The distinctive features of the inflammatory process in SCI include infiltration of monocyte-derived macrophages from the circulation to the lesion site. BMSCs-Exos can enhance the phagocytosis of macrophages and promote the phagocytosis of myelin debris by macrophages, thereby promoting functional recovery after SCI (Li et al., 2021a; Sheng et al., 2021). Type A2 astrocytes upregulate the expression level of some neurotrophic factors and thus play a protective role, whereas type A1 astrocytes form rapidly after injury and are toxic to myelin, synapses and neurons. The information-related nuclear factor-kappaB (NF-κB) pathway is associated with the activation of A1 astrocytes. BMSCs-Exos reduce the proportion of A1 astrocytes through the activation of transplanted NF-κB, which facilitates functional recovery after SCI (Wang et al., 2018). Moreover, in a recent study, it was shown that the expression levels of four pro-inflammatory factors TNF-α, IL-b, MCP-1, and MIP-1 were reduced, while the anti-inflammatory factors IL-10 and IL-4 were increased at the injury site after BMSCs-Exos transplantation, thus demonstrating their inflammation-suppressing effect after SCI (Fan et al., 2021).

The Wnt/β-catenin signaling pathway is a family of fully functional glycoproteins associated with pathophysiological processes such as cell proliferation, neurodevelopment, neuronal survival, and axon guidance. BMSCs-Exos promote tissue and functional repair of the spinal cord after injury and inhibit neuronal apoptosis by activating the Wnt/β-catenin signaling pathway. Bcl2, Bax and caspase family members are the main molecular components involved in regulating apoptosis (Gu et al., 2020). Bcl2 and Bax are common apoptotic markers of programmed cell death with anti-apoptotic and pro-apoptotic effects, respectively, and cleaved caspase has important roles in regulating programmed cell death. *In vitro* experiments revealed that BMSCs-Exos decreased the expression levels of caspase-3, caspase-9 and Bax proteins and increased the expression level of Bcl2 in primary neurons (Li et al., 2019). Sonic hedgehog (SHH) is a protein that carries exosomes and is important for organ and embryonic development in mammals (Jia et al., 2021a). The SHH signaling pathway is an important factor in neuronal regeneration after injury. A study found that after SCI, both BMSCs-Exos and BMSCs-SHH-Exos exerted some beneficial effects on functional recovery in rats, but the effect of BMSCs-SHH-Exos appeared to be more obvious, promoting increased neuronal functional recovery and inhibiting the activation of astrocytes to protect against injury-related pathology (Jia et al., 2021b).

Numerous studies show that miRNAs can regulate cell proliferation, differentiation, apoptosis, and are closely associated with pathological processes after SCI (e.g., inflammatory reaction, demyelination, oxidative stress, and neuronal apoptosis) (Wei et al., 2017). A study found that elevated miR-126 levels inhibited the inflammatory response, promoted vascularization, and improved functional recovery after SCI (Hu et al., 2015). MiRNAs are highly unstable, and must be delivered through an effective carrier system. Exosomes have emerged as a promising vehicle for miRNA delivery to the central nervous system (Pegtel et al., 2010; Sancho-Albero et al., 2019). MiRNA-126-modified BMSCs-Exos significantly inhibited the expression of Bax and caspase-3 and upregulated the expression of Bcl2 (Huang et al., 2020). BMSCs-Exos loaded with miRNA494 were shown to downregulate the expression of the astrocyte marker GFAP, while promoting an increase in neurofilaments (Huang et al., 2021a). Similarly, BMSCs-Exos injected with miRNA-29b were shown to decreased the expression of GFAP and concomitantly, increase the expression of the NF200 and GAP-43 in neurons following SCI (Yu et al., 2019). Injection of miRNA-133b-modified BMSCs-Exos through the tail vein of SCI rats was found to reduce the volume of the injury site, promote axonal regeneration, and activate ERK1/2, STAT3, and CREB, which are involved in neuronal survival and axonal regeneration (Li et al., 2018). After spinal cord injury, connective tissue growth factor (CTGF) is involved in the initial activation of injured glial cells, and overexpression of CTGF accelerates the proliferation of glial scar and hinders axonal migration and regeneration. Exos-siRNA was constructed by introducing a siRNA targeting CTGF into BMSCs-Exos through electroporation, which was then injected in to the tail vein of SCI rats. The results indicated that the siRNA could effectively inhibit GFAP expression, suppress the inflammatory response and neuronal apoptosis, and upregulate the levels of certain neurotrophic and anti-inflammatory factors, thus promoting the improvement of motor function (Huang et al., 2021b). Basic fibroblast growth factor (FGF2) plays a key role in cell differentiation and proliferation, angiogenesis, metabolism and tissue regeneration. Histone deacetylase 5 (HDAC5) is a target gene of miRNA-9-5p, and miRNA-9-5p from BMSCs-Exos was found to induce HDAC5 deacetylation-mediated FGF2 expression, thereby promoting functional recovery in SCI rats (He X. et al., 2022).

Spinal cord ischemia-reperfusion injury (SCIRI) frequently leads to neurological damage and death. High expression of BMSCs-Exos-loaded miRNA-124-3p was found to inhibit apoptosis, attenuate tissue and nerve damage caused by ischemia-reperfusion, and ameliorate SCIRI and related injuries by promoting M2 polarization (Li R. et al., 2020). Nogo-A, an axonal growth inhibitor, has inhibitory effects on neuronal cell migration and spreading and limits axonal regeneration after CNS injury. Nogo-A expression was upregulated after SCIRI, and expression of BMSCs-Exos-miRNA-455-5p downregulated Nogo-A and promoted motor function recovery (Liu B. et al., 2022).

#### 3.1.2 Human umbilical cord mesenchymal stem cell-derived exosomes (hucMSCs-Exos)

Human umbilical cord MSCs are the main cell population isolated from Wharton’s jelly, a gel tissue surrounding the umbilical vessels. They can negatively regulate antigen-presenting cells and T-cell apoptosis. Studies have shown that hucMSCs-Exos reduce apoptosis at the site of spinal cord injury, downregulate inflammatory factors and promote angiogenesis, axonal growth, and antifibrotic activity by activating the Wnt/β-linked protein pathway, while inhibiting microglia and astrocyte activation, thus playing an important role in promoting neuronal recovery (Rohde et al., 2019; Wang Y. et al., 2021; Kang and Guo, 2022). In addition, hucMSCs-Exos can promote the polarization of M1 macrophages to M2 macrophages (Sun et al., 2018). It was shown that hucMSCs-Exos reduced the expression of IL-1β and IL-6 in spinal cord tissue 24 h after SCI and reduced the deposition of type I collagen and NG2 at the lesion site.

#### 3.1.3 Human placental mesenchymal stem cell-derived exosomes (hPMSCs-Exos)

HPMSCs have a relatively high proliferative capacity and an advantage over other adult-derived stem cells in that they are usually disposed of as medical waste, and do not need to be obtained by invasive methods, allowing for large numbers with no related ethical issues. In addition, hPMSCs have good immunomodulatory, neuroprotective, and multidirectional differentiation properties. It was shown that intravenous administration of hPMSCs-Exos effectively increased the expression of the neural stem/progenitor cell marker SOX2^+^GFAP^+^PAX6^+^Nestin^+^cells in the spinal cord (Deng et al., 2021; Zhou et al., 2021). In addition, another study showed that hPMSCs-Exos promoted the tubular formation of endothelial cells *in vitro* and new vessel formation *in vivo*, and altered the recovery of sensory-motor function in SCI mice (Zhang et al., 2020).

#### 3.1.4 Other MSC-derived exosomes

In addition to the MSC-Exos mentioned above, studies have shown that human epidural adipose tissue mesenchymal stem cell-derived exosomes (ADSC-Exos) can modulate the inflammatory response (Sung et al., 2022). Moreover, exosomes secreted by ADSCs under hypoxia preconditioning are potential therapeutic agents in SCI that promote M1/M2 polarization of microglia (Shao et al., 2020). Meanwhile, exosomes derived from dental pulp stem cells have strong immunomodulatory effects, but their efficacy in promoting neurological and motor functions is unknown, as few studies have been conducted to address their potential (Liu C. et al., 2022). In our previous studies, we have described that human menstrual blood-derived mesenchymal stem cells (MenSCs), when combined with biomaterials, can provide an optimized bionic microenvironment for spinal cord injury repair, thereby reducing the inflammatory response, promoting neuronal differentiation and improving motor function. Therefore, we speculate that MenSCs-derived exosomes also have great potential in the treatment of spinal cord injury (He W. et al., 2022). Recent experiments on the use of exosomes derived from various cellular origins in SCI repair are shown in Table 1.

**TABLE 1:** Study of cell-derived exosomes in spinal cord injury.

Cell-derived exosomes	Dosage	Therapy schedule	Concentration	Methods of exosome isolation	Animal model	Results	References
BMSCs-Exos	200 μL	injected locally on the Surface of the injured Spinal cord	1 mg/mL	Immunomagnetic bead capture method	Contusion in mice	Promotes the recovery of neurological function after SCI by enhancing phagocytic ability of macrophages and promoting phagocytic ability of myelin debris through upregulation of macrophage collagenous structure of macrophage receptor (MARCO)	Sheng et al. (2021)
BMSCs-Exos	200 μL	tail vein injection	200 μg/mL	Ultracentrifugation	Contusion in rat	Effectively inhibits migration of pericytes, thereby maintaining the integrity of BSCB after spinal cord injury	Lu et al. (2019)
BMSCs-Exos	200 μL	tail vein injection	1 μg/µL	Ultrafiltration	Contusion in rat	Attenuating neuronal apoptosis by promoting early autophagy and facilitating functional behavioral recovery in spinal cord injured rats	Gu et al. (2020)
BMSCs-Exos	200 μL	tail vein injection	200 μg/mL	Ultracentrifugation	Contusion in rat	Protects pericytes by inhibiting prolapse and improving the integrity of the blood-spinal cord barrier, thereby promoting neuronal survival and nerve fiber extension	Zhou et al. (2022)
HucMSCs-Exos	200 μg	vein injection	1 μg/mL	Ultracentrifugation	Contusion in mice	Triggers polarization of macrophages from M1 to M2 phenotype, attenuates inflammatory response, and promotes recovery of motor function after spinal cord injury	Sun et al. (2018)
HucMSCs-Exos	200 μL	without providing Possible method	1 μg/µL	Ultrafiltration	Contusion in rat	Reduced activation of microglia and astrocytes in mice to improve motor function through anti-apoptotic and anti-inflammatory effects	Kang and Guo, (2022)
hPMSCs-Exos	200 μg/µL	injection	200 μg/mL	Ultracentrifugation	Contusion in mice	Pro-angiogenic effect on vascular endothelial cells	Zhang et al. (2020)
hPMSCs-Exos	100 μL	tail vein injection	500 μg/mL	Ultracentrifugation	Complete contusion in rat	Regulates endogenous neural precursor cells, promotes nerve regeneration, and facilitates functional recovery after spinal cord injury	Zhou et al. (2021)
hEpi AD-MSC exosome	200 μL	vein injection	Low:1 × 10^9^particles/0.2 mL; High:5 × 10^9^particles/0.2 mL	Ultrafiltration	a 50 g clip-compression in rat	Restoring spinal cord function by reducing the inflammatory response	Sung et al. (2022)
NSCs-Exos	100 μL	tail vein injection	2 mg/mL	Immunomagnetic bead capture method	Moderate contusion in mice	Introduction of VEGF-A into SCMECs promote the angiogenic activity of SCMECs, and NSCs-Exos treatment promotes the recovery of neurological function after spinal cord injury, which is largely attributed to its pro-angiogenic effect	Zhong et al. (2020)
NSCs-Exos	200 μL	tail vein injection	1 μg/µL	Ultrafiltration	Contusion in rat	Effectively reduce apoptosis and neuroinflammation of nerve cells after spinal cord injury, thus promoting functional recovery after spinal cord injury	Rong et al. (2019)
SCDEs	250 μL	tail vein injection	0.1 μg/µL	Ultracentrifugation	Contusion in rat	Inducing axonal protection after spinal cord injury by increasing autophagy and reducing apoptosis	Pan et al. (2022)
SCDEs	100 μL	tail vein injection	0.1 μg/µL	Ultracentrifugation	Severe crush	Reducing CSPG deposition through NF-κB/PI3K signaling pathway by increasing TLR2 expression on astrocytes promotes functional recovery after spinal cord injury in mice	Pan et al. (2021))
Macrophage-derived Exosome	100 μL	tail vein injection	100 μg/µL	Ultracentrifugation	Moderate contusion in mice	Activation of Wnt/OTULIN-catenin signaling pathway through delivery of beta protein positively regulates vascular regeneration and neurological recovery after spinal cord injury	Luo et al. (2021)
MG-Exos	200 μL	tail vein injection	1 μg/µL	Ultracentrifugation	Moderate contusion in mice	Protects spinal microvascular endothelial cells from the toxic effects of high oxidative stress and promotes endothelial cell function and viability	Peng et al. (2021)
Pericyte-derived exosome	300 μL	tail vein injection	200μg/3 mL	Ultracentrifugation	Contusion in mice	Promotes blood flow after spinal cord injury, improves endothelial function, protects the BSCB, and attenuates apoptotic responses, thereby promoting functional and behavioral recovery	Yuan et al. (2019)

### 3.2 Neural stem cell-derived exosomes (NSCs-Exos)

NSCs-derived exosomes carry bioactive RNA, proteins, lipids and other substances, and regulate synapses and maintain vascular integrity. NSCs-Exos have the following advantages in the treatment of SCI over the NSCs: 1) as they are unable to replicate *in vivo* and will disintegrate quickly, there is almost no malignant transformation; and 2) because of their small size, they are less likely to cause small vessel obstruction and can cross the blood-spinal cord barrier through intravenous injection. Studies have shown that NSCs-Exos can promote angiogenesis of spinal microvascular endothelium (SCMEC), reduce spinal cord cavities, and promote motor function recovery, thus facilitating SCI repair (Zhong et al., 2020); FTY720 is a functional antagonist of sphingosine 1 phosphate receptor-1 (S1P1), which participates in immune regulation. As evidence has indicated that systemic FTY720 application can cause adverse effects, NSCs-Exos were loaded with FTY720 (FTY720-NSCs-Exos) were injected into the tail veins of SCI mice after SCI and changes in behavior, inflammatory factors, and neuronal apoptosis were assessed. The results indicated that FTY720-NSCs-Exos alleviated pathological changes and improved motor function in the hind limbs of the mice. Meanwhile, *in vitro* studies revealed that FTY720-NSCs-Exos had a protective effect on the SCMEC barrier in a hypoxic environment (Chen et al., 2021).

### 3.3 Schwann cell-derived exosomes (SCDEs)

SCDEs can stimulate the expression of Toll-like receptors (TLR2) in astrocytes after SCI and reduce the deposition of CSPGs through NF-κB/PI3K signaling, thereby promoting functional recovery after SCI in mice (Pan et al., 2021). SCDEs promoted the regeneration of axonal growth, and sequencing results indicated that SCDEs released from the areas of axonal injury contained miRNAs associated with regeneration (Zhou et al., 2018). Moreover, SCDEs increased autophagy after SCI and reduce PTEN activity by activating vincristine receptor B signaling, thereby reducing apoptosis and promoting recovery of motor function (Pan et al., 2022).

### 3.4 Macrophage-derived exosomes

Accumulating evidence suggests that M1 macrophages produce large amounts of pro-inflammatory cytokines, which aggravate the damage in the injured spinal cord, whereas the M2 phenotype produces anti-inflammatory factors that are thought to have neuroprotective effects. Angiogenesis after SCI is extremely important in the recovery process. Protein expression profiles in M2 macrophages and M2-Exos were analyzed using iTRAQ technology, and GO analysis was performed on the top 20 upregulated proteins out of 307 differential proteins identified. Among them, OTULIN was the most significantly changed protein and expressed at 6.81-fold higher levels in M2-Exos than in M2 macrophages. OTULIN protein in M2-Exos promotes angiogenesis after SCI by activating the Wnt/β-Catenin signaling pathway and triggering angiogenesis-related gene expression in spinal microvascular endothelial cells (Luo et al., 2021). Peripheral macrophage-derived exosomes (PM-Exos) may play an important role in the anti-inflammatory process by activating microglia autophagy through inhibition of the PI3K/AKT/mTOR signaling pathway (Zhang B. et al., 2021).

After spinal cord injury, microglia release inflammatory factors. The M1 and M2 phenotypes have opposing roles in the activation of macrophages/microglia, and can reduce the inflammatory response by promoting the polarization of the M1 phenotype to the M2 phenotype. Berberine, derived from the Chinese herbal medicine huanglian, has pharmacological effects such as anti-inflammatory, antioxidant, neuroprotective, and anti-microbial. Loading berberine into M2-type primary macrophage-derived exosomes as a novel drug delivery system for the treatment of SCI can effectively overcome traditional disadvantages of other molecules such as the low efficiency of crossing the blood-spinal cord barrier. It was also demonstrated that Exos-berberine significantly decreased the expression of the M1 marker proteins iNOs and CD86 and increased the expression of the M2 marker protein CD206, which indicated that Exos-berberine had better inhibitory effects on inflammation, reduced neuronal apoptosis, and promoted motor function recovery. The bioavailability of Exos-berberine was upregulated by 3.2-fold compared to berberine solution alone (Gao et al., 2021). In another study, the natural polyphenol curcumin (Cur) which has anti-inflammatory properties, was loaded to a matrix metalloproteinase 9 (MMP) cleavable linker (CL) containing the rVGLP sequence, and used to couple nerve growth factor to the surface of exosomes to prepare Cur@EVs^−CL-NGF^ for the treatment of SCI. The Cur@EVs^−CL-NGF^ had the advantages of 1) prolonging the circulation time of NGF *in vivo*; 2) NGF dissociating and being released into the microenvironment over time; 3) overcoming the disadvantages of curcumin’s own low solubility and biological half-life; and 4) strong inhibition of M1 macrophages (Zhang C. et al., 2021).

### 3.5 Microglia-derived exosomes (MG-exos)

Microglia are a type of neuroglia, equivalent to macrophages in the brain and spinal cord. Microglia and macrophages are considered to be important players in the pathophysiology of spinal cord injury (Poulen et al., 2021). miR-151-3p is highly expressed in MG-Exos and plays a neuroprotective role during the repair of SCI. Meanwhile, MG-Exos can activate the p53/p21/CDK1 signaling cascade to regulate neuronal apoptosis and promote axonal growth (Li et al., 2021b). MG-Exos may act as an antioxidant through activation of the Keap1/Nrf2/HO-1 pathway to promote functional recovery after SCI (Peng et al., 2021).

### 3.6 Pericyte-derived exosomes

Pericytes surround the endothelial cells of capillaries and small veins that are located throughout the body and are important components of the neurovascular unit (Yuan et al., 2019). After SCI, pericytes play an important role in the regulation of capillary tone and spinal cord blood flow (Li et al., 2017). Also, the degree of pericyte-dependent vasoconstriction in patients with spinal cord injury varies depending on the degree of injury (Almeida et al., 2018). Pericytes are equally important for vascular development, maturation, permeability, and maintenance of the integrity of the blood-spinal cord barrier (Picoli et al., 2019). The glial scar tissue formed by subpopulations of perivascular cells, called type a pericytes, is extremely important for restoring tissue integrity and is a target for the development of therapies to promote axonal regeneration after CNS injury (Dias et al., 2018). Due to the close relationship between pericytes and endothelial cells, pericyte-derived exosomes are more likely to be taken in by endothelial cells, and are involved in the regulation of endothelial function. It was found that pericyte-derived exosomes have an irreplaceable role in alleviating pathological changes and improving motor function after SCI, and can protect the blood-spinal cord barrier, reduce edema, and inhibit apoptosis. *In vitro* experiments demonstrated that exosomes improve endothelial barrier function under hypoxic conditions and protect endothelial cells through the PTEN/Akt pathway (Yuan et al., 2019).

In the process of SCI repair, cell-derived exosomes are an extremely beneficial factor in the treatment of SCI, whereas, direct injection of cell-derived exosomes into the SCI site has some limitations: 1) the most common way to delivery exosomes into the sites of injury is *via* local injection, nevertheless, this may lead to rapid clearance of exosomes from the site (Brennan et al., 2020; Murali and Holmes, 2021); 2) the ischemic and hypoxic environment at the injury site inevitably causes deterioration of the injected exosomes (Fan et al., 2018). Biomaterial scaffolds play a crucial role in building a microenvironment conducive to regeneration (Liu S. et al., 2021). Therefore, biomaterial scaffold transplantation is a key strategy for SCI repair because biomaterial scaffolds not only provide guidance for nerve regeneration after SCI, but also deliver and fix substances such as exosomes, cells or other neurotrophic factors to the injury site (Tang et al., 2021). At the same time, the mechanism of biomaterial scaffolds to promote motor function recovery relies on bridging the connection to the spinal cord stump rather than the long-distance regeneration of ascending sensory axons and descending motor axons (Yang et al., 2020).

## 4 Various forms of biomaterial scaffolds

Biomaterial scaffolds should have properties such as appropriate porosity, permeability, and good cytocompatibility (Liu et al., 2019), and provide support for migration, adhesion, proliferation and differentiation of neuronal cells (Shen et al., 2021). Furthermore, biodegradability, low immunogenicity, simple design (Katoh et al., 2019), and the ability to provide structural support for axonal regeneration are all equally important attributes (Liu et al., 2017). Biomaterials have promising applications in enhancing spinal cord injury repair (Courtine and Sofroniew, 2019). Several types of biomaterials have been applied in tissue engineering, which is mostly prepared from natural and synthetic polymers and can be classified into three main forms: hydrogel, 3D printing, and nanomaterials.

### 4.1 Hydrogel

Hydrogel is a polymeric material with high water content and a variety of physical properties. Hydrogels are 3D networks of cross-linked hydrophilic polymer chains that can be designed into almost any shape and size to resemble human tissue. There are four main properties for biomedical applications: degradability, adhesion, bioactivity, and mechanical properties (Seliktar, 2012). Hydrogels are ideal scaffolds for the treatment of spinal cord contusions because they can be injected into the irregular shape of the contused spinal cord and can mimic the mechanical properties of natural spinal cord tissue. Most hydrogels exhibit tunable mechanical properties that simulate those of soft tissues in the central nervous system (McKay et al., 2014). Hydrogels can provide a conducive environment for transplanted cells with high elasticity, high permeability, non-toxicity, and good mechanical properties. Hydrogels can also provide support for the injured spinal cord, form a local bridge for nerve regeneration and prevent scar formation, and the 3D porous structure provides a matrix for supporting tissue implantation (Tsintou et al., 2015). Currently, hydrogels used for spinal cord injury repair include natural hydrogels such as alginate, agarose, collagen, fibronectin, gelatin, and ECM, as well as synthetic hydrogels such as polylactic acid, polylactic acid-ethanolic acid, and polyethylene glycol (Hou et al., 2022).

The extracellular matrix contains intrinsic biochemical and mechanical signals that play an irreplaceable role in regulating the phenotype and function of cell development, homeostasis, and response to injury (Hussey et al., 2018). On the one hand, it serves as the supporting structure, on the other hand, it can maintain the activity of exosomes. The ECM currently used in preclinical models mainly includes spinal cord, brain, muscle, bone, tendon, heart, liver, lung, and fat (Spang and Christman, 2018). After spinal cord contusion in rats, application of decellularized extracellular matrix (dECM) hydrogel modulated M1 type macrophage polarization toward the M2 type, reduced the size of the injury gap, and promoted motor function recovery up to 8 weeks post-injury (Hong et al., 2020). Moreover, hucMSCs encapsulated in hydrogels secrete chemokines that are associated with neuronal protection by reducing N-methyl-D-aspartate (NMDA) receptor expression and extracellular glutamate levels to protect neurons from excitotoxicity and/or contribute to the survival of neuronal proteins in axons and promote locomotion (Papa et al., 2018).

In a study by Dai et al., collagen scaffolds loaded with mononuclear cells from the patient’s own bone marrow or hucMSCs were transplanted into SCI patients. Some patients with acute or chronic complete spinal cord injury have improved sensory and motor function, and some even regain the ability to walk on their own. The findings suggest that functional stent transplantation may be an irreplaceable therapy for patients with complete spinal cord injury (Tang et al., 2021). This study is registered with ClinicalTrials.gov: NCT02688049. A new agarose/carbomer-based hydrogel combines different strategies to optimize the viability, density, and paracrine factor transport of hucMSCs. Combining arginine-glycine-aspartic acid tripeptide and 3D extracellular matrix deposition increase the ability to attach and maintain hucMSCs in the hydrogel. This was ultimately shown to significantly immunomodulate the pro-inflammatory environment and increase the number of M2 macrophages in a mouse SCI model (Caron et al., 2016). Similarly, a study compared decellularized matrix hydrogels derived from the spinal cord (DSCM-gel) and peripheral nerves (DNM-gel). DSCM-Gel had a higher porosity than DNM-gel, which promoted the proliferation and migration of neural stem/progenitor cell (NSPC) in 3D culturing, followed by facilitation of the NSPCs differentiation into neurons. *In vivo*, the number of Nestin^+^/BLBP^+^ cells and Nestin^+^/β-Tubulin III^+^ cells were higher in the DSCM-gel, compared with the DNM-gel 14 days after implantation, indicating that the implanted DSCM-gel effectively promoted the differentiation of endogenous NSPC into neurons rather than glial cells (Xu et al., 2021).

### 4.2 3D printing

The combination of 3D printing technology and tissue engineering has opened up new research areas for neurological reconstruction after spinal cord injury. 3D bioprinting technology is a technology based on biomaterial scaffolds, cells and other biologically active molecules, which can accurately and efficiently simulate complex and personalized bionic functional scaffolds. 3D bioprinting technology has been applied to a variety of tissues including bone, cartilage, vascular systems, muscle and heart, but there are relatively few studies regarding SCI (Koffler et al., 2019; Sun et al., 2019; Hou et al., 2022).

An ideal 3D-printed scaffold requires not only a high-density porous structure to bridge and direct regenerative axonal growth, but also good mechanical properties. It was reported that implantation of 3D-printed collagen-chitosan scaffolds promoted axonal regeneration and neurological function recovery and reduced glia scar and cavity formation in rats compared to conventionally prepared collagen-chitosan scaffolds (Sun et al., 2019). Both natural and artificial materials can be used to construct 3D-printed scaffolds. Type I collagen, a natural material that is the main component of the ECM, is widely used in 3D culture systems due to its good biocompatibility, mechanical strength, degradability, and immunogenicity. The traditional 2D stem cell culture system cannot simulate the endogenous microenvironment. In contrast, the stem cells cultured in the 3D system show a more accurate cell behavior model in terms of cell attachment, viability, self-renewal, migration, and differentiation (Baharvand et al., 2006). Furthermore, 3D cultured cells exhibited different gene expression and provided different cell polarization compared to 2D cultured cells (Pampaloni et al., 2007; Caron et al., 2016). In one study, a neural progenitor cells-encapsulated 3D bionic scaffold was applied to create a complex central nervous system structure for the repair of SCI. It was shown that injured host axons regenerated and grew into the 3D bionic scaffold and out from the lesion site into the distal host spinal cord. Bioprinting uses living cells and biomaterials to design 3D tissue structures with precisely defined structures and geometries. Bioink is an important part of bioprinting and is usually composed of biomaterials (such as hydrogels), cells or cell aggregates, or a combination. Several natural polymers (such as alginate and gelatin) and synthetic polymers have been used as bioinks (Gungor-Ozkerim et al., 2018). In a study, a collagen/heparin sulfate scaffold constructed with a 3D bioprinter was shown to enhance the mechanical properties of collagen and provide continuous guide channels for axons, thereby improving neurological function after SCI. This study showed an increase in the number of neurofilament positive cells in the collagen/heparin sulfate 3D scaffold group compared to the collagen group. At the same time, the motor function and the outcome of electrophysiological experiment were significantly recovered in animal SCI models (Chen et al., 2017).

### 4.3 Nanomaterials

Recent developments in various nano-strategies offer effective ways to cross the blood-spinal cord barrier and provide therapeutic agents after SCI. Biomaterials containing nanoscale fibers can increase axonal extension and proliferation of neural stem cells *in vitro* compared to micron-sized fibers. The main nanomaterials applied in SCI repair are nanoparticles, nanofibers, carbon nanotubes, and quantum dots. The delivery strategy is mainly through targeting the inflammatory response, removing inhibitory components and promoting axonal regeneration. Among them, quantum dots and carbon nanotubes have great potential in spinal cord injury repair due to their small size, which can promote extravasation and systemic clearance of the blood-spinal cord barrier and maintain high neurobiological activity after cellular uptake (Song et al., 2019). Studies have demonstrated that minocycline nanoparticles, when given acutely in a mouse SCI model, can effectively modulate resident microglia, reduce inflammatory responses, and maintain a conducive environment for regeneration (Papa et al., 2016). Either alone or in combination with hydrogels, electrospun nanofiber-guided channels are very promising. Among them, electrospinning is more advantageous due to its ease of fabrication. Nanofibers provide a 3D network that is more conducive to cell attachment, migration, proliferation and differentiation than conventional scaffolds. In terms of morphology and diameter, the fibers have similarities to natural ECM and thus can provide a conducive environment for neural regeneration (Tsintou et al., 2015). Peptide amphiphile nanofibers (PAs) are a promising class of molecules that can mimic the regulatory properties of the natural environment of cells. Recently, self-assembled PAs showed promising results in regard to neural differentiation *in vitro* and neural regeneration *in vivo*, demonstrating the potential of these materials for effective treatment of CNS injuries (Sever-Bahcekapili et al., 2021).

## 5 Exosomes combined with biomaterial scaffolds in spinal cord injury

Exosomes combined with biomaterial scaffolds have shown good therapeutic effects in research related to tissue regeneration and angiogenesis (Table 2) (Lazar et al., 2021). Due to certain limitations of stem cell-derived exosomes in the treatment of spinal cord injury, research has focused on the application of exosomes combined with biomaterial scaffolds in order to improve the retention of exosomes in the target site. Biomaterials such as hyaluronic acid hydrogel and alginate, have been used to deliver cell-derived exosomes for tissue regeneration, which allowed for sustained and controlled release to improve bioavailability (Brennan et al., 2020) and enhance therapeutic efficacy (Wang et al., 2019; Yuan et al., 2020; Murali and Holmes, 2021). These biomaterial scaffolds, as alternatives to ECM, need to meet the following: 1) maintain exosomes at the site of injury and preserve their properties and structural characteristics; 2) release exosomes into the ECM for a sufficiently long period of time; and 3) bind to the injured tissue and support the migration of neighboring cells into the scaffold (Liu et al., 2019; Poongodi et al., 2021).

**TABLE 2:** Study on the treatment of spinal cord injury by cell-derived exosomes combined with biomaterial scaffolds.

Bio-scaffold	Cell-derived exosomes	Animal model	Results	References
Fibrin glue (FG)	BMSCs-Exos	Contusion in rat	Reduces oxidative and inflammatory microenvironment, promotes nerve tissue repair and urinary tissue protection	Mu et al. (2021)
GelMA	BMSCs-Exos	Transection in rat	Promotes neuronal differentiation and extension *in vitro*, facilitates nerve regeneration, and reduces glial scarring at damaged injury sites	Cheng et al. (2021)
PPFLMLLKGSTR peptide modified HA hydrogel (PGel)	hPMSCs-Exos	Transection in rat	Reduces inflammation and oxidation, significantly restores nerves and preserves urinary tissue	Li R. et al. (2020)
F127-polycitrate-polyethyleneimine hydrogel (FE)	ADMSCs-Exos	Transection in rat	Reduces inflammatory response, remyelination and axonal regeneration, and significantly promotes tissue repair and recovery of motor function after spinal cord injury	Wang Y. et al. (2021)
Adhesive HA hydrogel	hypo- HUVECs	Transection in rat	Promotes angiogenesis and nerve regeneration at the site of spinal cord injury	Mu et al. (2022)
Melatonin (MT)	BMSCs	Contusion in mice	Crossing the blood-brain barrier for spinal cord injury	Liu W. et al. (2021)
LOCS-BSP-PTX	hucMSCs-Exos	Transection in rat	Guided axonal growth along its fibers *in vitro* reduces the deposition of scar-associated components at the injury site and promotes complete spinal cord injury in rats	Zhang L. et al. (2021)
GM/PPy	BMSCs	Right hemisection in mice	Promoting NSC recruitment, neuronal and myelin-associated axon regeneration and synergistic motor recovery after spinal cord hemisection in mice	Fan et al. (2022)

A local implantation of human placental amniotic mesenchymal stem cell-derived exosomes (hPMSCs-Exos) based on the PPFLMLLKGSTR peptide-modified hyaluronic acid hydrogel (pGel) was found to increase the spinal cord cavity and promote motor function of hind limb in rats (Li L. et al., 2020). Hypoxia-stimulated exosomes (Hypo-Exo) were transplanted to the injury site in a peptide-modified adhesive HA hydrogel package to promote angiogenesis after SCI. After hypoxic stimulation, hypoxia-inducible factor 1-alpha (HIF-1α) expression was upregulated in human umbilical cord blood MSC-derived exosomes. Thus, the adherent hydrogel system (hypoExo@HA) significantly improved angiogenesis, neuroregeneration, and functional recovery. The use of exosomes in the treatment of spinal cord injury under hypoxic stimulation was shown to have great potential (Mu et al., 2022). Specifically, a spongy alginate scaffold combined with human umbilical cord mesenchymal stem cell exosomes (hucMSCs-Exos) was developed as a therapy for pain caused by L5/6 spinal nerve ligation (SNL). The analgesic effect was found to be detectable at 24 h after implantation, with almost complete elimination of SNL-induced injury the next day. The analgesic effect lasted until 21 days after ligation, indicating that the graft had long-lasting effects (Hsu et al., 2020). An injectable adhesive anti-inflammatory F127-polycitrate-polyethyleneimine hydrogel (FE), with sustainable and long-term release of exosomes encapsulated on the injured spinal cord, was shown to inhibit glia scar formation and inflammation, and promote axonal regeneration (Wang C. et al., 2021). Since most SCI events occur away from hospital and cannot be treated in time, it is necessary for interventions in the microenvironment of the lesion site to be conducted at the initial stage, otherwise secondary damage will occur. The *in situ* formation of fibrin glue utilizes fibrinogen and thrombin from natural human blood as two gelation constituents to mimic the blood clotting process as a viable emergency treatment. Peripherally encapsulated fibrin glue combined with exosomes (FG-Exos) reduced oxidation and inflammation (Mu et al., 2021). A composite hydrogel system containing exosomes (GelMA-Exos), was synthesized using UV light and into a 3D hydrogel to promote axonal growth compared to treatment with exosomes alone (Cheng et al., 2021). It was shown that silk fibroin (SF) had excellent mechanical properties and silk sericin (SS) induced adhesion and biocompatibility. Human umbilical cord blood MSC-derived exosomes encapsulated in SF-SS hydrogels promoted nerve growth and suppressed the inflammatory response (Han et al., 2021), and we speculate that they have potential in SCI repair.

**FIGURE 2:**
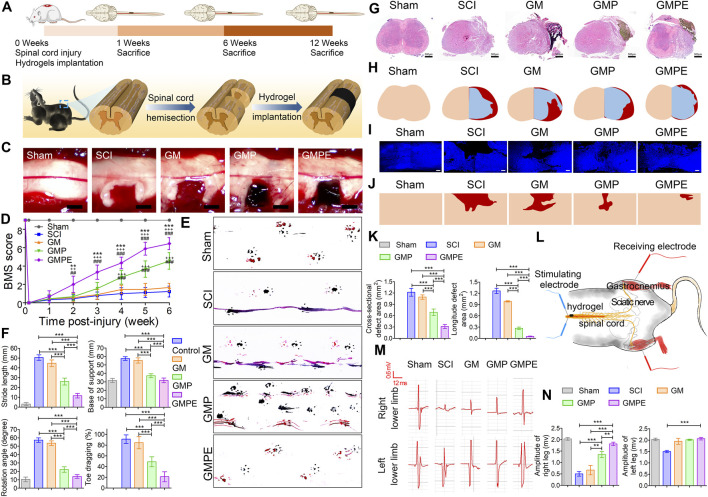
Functional recovery of mice in each subgroup. **(A)** Schematic diagram of the experimental timeline. **(B)** Demonstration of spinal cord hemisection and hydrogel implantation. **(C)** Implantation of different hydrogels in the cavity site. **(D)** BMS score to assess motor recovery of the right hind limb in mice. Compared to GMP (*p < 0.05, **p < 0.01, ***p < 0.001) and GM (+ *p* < 0.05, ++ *p* < 0.01, +++ *p* < 0.001) hydrogels and SCI (#*p* < 0.05, ##*p* < 0.01, ###*p* < 0.001), GMPE hydrogel-treated mice had improved BMS motor scores (n = 9). **(E)** Recovery of motor function was assessed by footprints. Blue represents forelimb footprints and red represents hindlimb footprints. **(F)** Step length, point of support, rotation angle and toe drag were quantified to assess motor recovery 6 weeks post-injury (n = 9). **(G)** HE staining showing the morphology of transverse spinal cord sections after hydrogel implantation. **(H)** Representative reconstruction of the spinal cord cross-sections. The color of normal tissue was fresh, the color of cavity was red, and the color of regenerated tissue was blue, respectively. **(I)** Immunofluorescence images demonstrating the morphology of longitudinal sections of the spinal cord. **(J)** Images of reconstructed longitudinal sections of the spinal cord. Skin tones and red areas represented normal tissue and cavity areas, respectively. **(K)** Quantification of cavity volumes in transverse and longitudinal sections of the spinal cord (n = 3). **(L)** CMAPs testing protocol for mice in the GMPE hydrogel treatment group was shown. **(M)** CMAPs results for mice in the normal group and various hydrogels implanted for 6 weeks. **(N)** Quantification of the CMAPs amplitudes measured in mice of the sham and hydrogel treatment groups (n = 3). Reprinted with permission from Fan,L.; Liu,C.; Chen,X.; Zheng,L.; Zou,Y.; Wen,H.; Guan,P.; Lu,F.; Luo,Y.; Tan,G.; Yu,P.; Chen,D.; Deng,C.; Sun,Y.; Zhou,L.; Ning,C. (2022). Copyright 2022 Advanced Science, Wiley-VCH GmbH (Fan et al., 2022).

Studies have demonstrated that melatonin is isolated from the pineal gland and is widely distributed throughout the body. Melatonin-pretreated MSCs-Exos can polarize M1 type microglia/macrophages to M2 type and better promote functional recovery in SCI mice (Liu W. et al., 2021). By intranasal administration, the exosomes can cross the blood-brain barrier and are better retained at the site of injury than administered intravenously (Guo et al., 2019). Using human umbilical cord MSC-derived exosomes (MExos) as drug carriers, a MExos collagen scaffold was designed by a novel dual biospecific peptide (BSP) with the ability to promote neural stem cell migration and paclitaxel (PTX) delivery. As a major component of the extracellular matrix, collagen has better properties than other materials in promoting cell adhesion and growth. Linearly ordered collagen scaffolds (LOCS) and PTX were selected to construct multifunctional collagen scaffolds (LOCS-BSP-MExos-PTX, LBMP) for repairing complete transected SCI rats, by promoting nerve regeneration, reducing scar deposition, and facilitating functional recovery (Zhang L. et al., 2021). Conductive hydrogels are an attractive candidate to promote SCI repair due to their electrical and mechanical properties matching those of neural tissues, and a double-networked conductive hydrogel composed of photo-cross-linked gelatin methacrylate (GM) hydrogel and polypyrrole (PPy) hydrogel was developed to form a GM/PPy/exosomes (GMPE) hydrogel. It can reduce CD68-positive microglia early after SCI. *In vitro* and *in vivo* studies revealed that GMPE hydrogels promote the differentiation of neural stem cells to neurons and oligodendrocytes and promote axonal growth *via* conductive hydrogels that co-activate the PTEN/PI3K/AKT/mTOR pathway (Figure 2) (Fan et al., 2022).

## 6 Prospects and challenges for exosomes combined with biomaterial scaffolds

Due to the complexity of the pathological microenvironment of spinal cord injury, there is no fully effective treatment method. At present, research for exosomes combined with biomaterial scaffolds for SCI is a promising approach, but it is still in the exploratory stage and there are not enough related studies. Also there is no uniform standard for the specific time of graft transplantation after injury. There is no unified international standard for the purification of exosomes and the safety and therapeutic efficacy of stents, which is also an urgent issue to be addressed. Larger animals with longer lifespans should be selected to improve the assessment of graft risk when evaluating treatment outcomes, and SCI models should include all types of injury over time to bring preclinical models closer to clinical models.

## 7 Conclusion

Biomaterials provide a supportive microenvironment while exosomes continuously release growth factors and other nutrients. We believe that safer biomaterial scaffolds will be generated to improve the efficiency of SCI treatment. In the future, purification techniques for exosomes will be further improved. In conclusion, cell-derived exosomes combined with biomaterials play a role in the treatment of SCI.
